# A-WINGS: an integrated genome database for *Pleurocybella porrigens* (Angel’s wing oyster mushroom, Sugihiratake)

**DOI:** 10.1186/1756-0500-7-866

**Published:** 2014-12-03

**Authors:** Naoki Yamamoto, Tomohiro Suzuki, Masaaki Kobayashi, Hideo Dohra, Yohei Sasaki, Hirofumi Hirai, Koji Yokoyama, Hirokazu Kawagishi, Kentaro Yano

**Affiliations:** Bioinformatics Laboratory, School of Agriculture, Meiji University, 1-1-1 Higashi-Mita, Kawasaki, 214-8571 Japan; Research Institute of Green Science and Technology, Shizuoka University, 836 Ohya, Suruga-ku, Shizuoka, 422-8529 Japan; Department of Applied Biological Chemistry, Faculty of Agriculture, Shizuoka University, 836 Ohya, Suruga-ku, Shizuoka, 422-8529 Japan; Graduate School of Science and Technology, Shizuoka University, 836 Ohya, Suruga-ku, Shizuoka, 422-8529 Japan

**Keywords:** Angel’s wing, Transcriptome, Genome, Database, Gene expression

## Abstract

**Background:**

The angel’s wing oyster mushroom (*Pleurocybella porrigens*, Sugihiratake) is a well-known delicacy. However, its potential risk in acute encephalopathy was recently revealed by a food poisoning incident. To disclose the genes underlying the accident and provide mechanistic insight, we seek to develop an information infrastructure containing omics data. In our previous work, we sequenced the genome and transcriptome using next-generation sequencing techniques. The next step in achieving our goal is to develop a web database to facilitate the efficient mining of large-scale omics data and identification of genes specifically expressed in the mushroom.

**Findings:**

This paper introduces a web database A-WINGS (http://bioinf.mind.meiji.ac.jp/a-wings/) that provides integrated genomic and transcriptomic information for the angel’s wing oyster mushroom. The database contains structure and functional annotations of transcripts and gene expressions. Functional annotations contain information on homologous sequences from NCBI nr and UniProt, Gene Ontology, and KEGG Orthology. Digital gene expression profiles were derived from RNA sequencing (RNA-seq) analysis in the fruiting bodies and mycelia. The omics information stored in the database is freely accessible through interactive and graphical interfaces by search functions that include ‘GO TREE VIEW’ browsing, keyword searches, and BLAST searches.

**Conclusions:**

The A-WINGS database will accelerate omics studies on specific aspects of the angel’s wing oyster mushroom and the family *Tricholomataceae*.

## Findings

The angel’s wing oyster mushroom (*Pleurocybella porrigens*, Sugihiratake in Japanese) belongs to the family *Tricholomataceae*[1]. The genus *Pleurocybella* is monotypic, and contains only the angel’s wing oyster mushroom, which is widespread in temperate forests of the northern hemisphere [2]. The fruiting body is widely recognized as a delicacy, and is consumed all over the world. Unexpectedly, in 2004, a total of 55 cases of food poisoning were reported due to consumption of the mushroom, with 17 of them ending in death due to acute encephalopathy. Despite an aggressive effort to identify the underlying cause of these events [3–11], the molecular basis remains obscure.

To disclose the genes underlying the poisonings and provide mechanistic insight, we have sought to develop an information infrastructure containing omics data. In a previous study, we sequenced the genome and transcriptome of the mushroom using next-generation sequencing techniques [12]. That study revealed that, compared to other Agaricales, the genome of the angel’s wing had a unique structure and contained numerous novel genes. Deep mRNA sequencing of the fruiting bodies and mycelia revealed the existence of 45,390 and 26,216 unigenes (non-redundant sequence sets for expressed genes), respectively. Based on the obtained sequences, we identified differentially expressed genes between the fruiting bodies and mycelia. Given this genomic and transcriptomic data, we believed that a web database would facilitate the efficient mining of the large-scale omics data and identification of genes specifically expressed in the mushroom.

This study introduces the web database A-WINGS (http://bioinf.mind.meiji.ac.jp/a-wings/), which provides integrated genomic and transcriptomic information on the angel’s wing oyster mushroom. The database contains structure and functional annotations of transcripts and gene expressions. The functional annotations include information on: (i) homologous sequences detected by BLAST sequence similarity searches [13] against the non-redundant protein database (nr) in the NCBI [14] and universal protein knowledgebase (UniProt) [15]; (ii) Gene Ontology (GO) terms [16] obtained by InterProScan [17]; and (iii) KEGG Orthology [18]. Digital gene expression profiles were derived from RNA sequencing (RNA-seq) analysis in the fruiting bodies and mycelia, respectively. The omics information stored in the database is freely accessible through interactive and graphical interfaces by search functions including ‘GO Tree View’ browsing, keyword search, and BLAST search.

**Figure 1:**
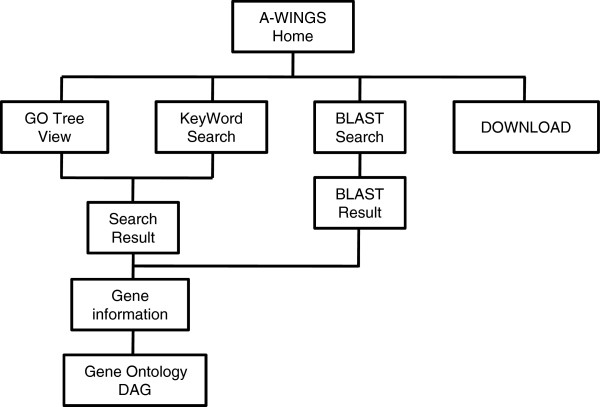
**The database structure of A-WINGS.** The omics data are accessible through the search functions in tab-based interfaces. The graphical, interactive web interfaces allow users seamless access to information available in A-WINGS.

**Figure 2:**
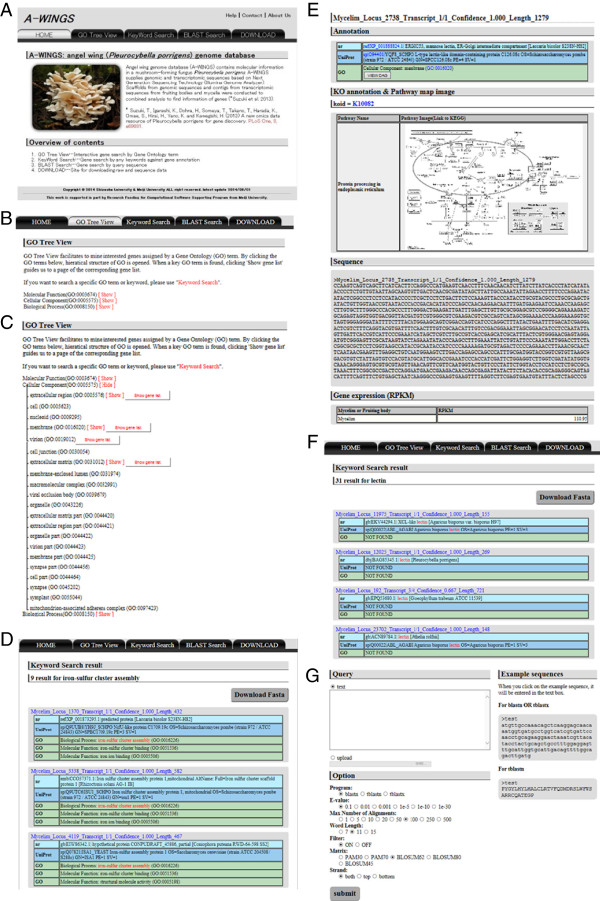
**Graphical user interfaces of A-WINGS: (A) top (parent) page; (B) GO Tree View page; (C) GO Tree View page with ‘child’ GO terms; (D) the gene list associated with a GO term; (E) the detailed page for unigene (transcript information); (F) search results obtained by the keyword search function; (G) the BLAST search interface.**

A-WINGS was developed by the LAMP (Linux, Apache, MySQL and Perl) stack. We stored omics data of the angel's wing [12] into MySQL database (version 5.0.95) on an Apache HTTP server (version 2.2.27) running on a Linux operating system (CentOS release 5.11, 64-bit). We then developed an interactive and graphical web-interface written in PHP and HTML for the data access. The database was equipped with three search functions—Keyword Search, GO Tree View and BLAST Search—provided by tab-based interfaces on the web page (Figures 1 and 2A).

The GO Tree View is an interactive and graphical viewer for effective browsing of Gene Ontology (GO) terms [16] and gene (unigene) lists. GO terms and GO identifiers are presented in directed acyclic graph (DAG) [16]. When users access the GO Tree View, the top three categories (i.e., Cellular Component [GO:0003674], Biological Process [GO:0005575], and Molecular Function [GO:0008150]) appear in the viewer (Figure 2B). Clicking ‘parent’ GO terms with a broader sense, calls up ‘child’ terms with a narrower sense (more specialized terms) (Figure 2C). The gene list associated with a GO term is also accessible by clicking ‘Show gene list’ (Figure 2D).

The list of genes (unigenes) includes information on unigene names and brief functional annotations. The functional annotations contain homologous sequences in the nr, UniProt, and GO terms. Clicking on the hyperlink of a unigene retrieves detailed information on the unigene (Figure 2E). In addition to the brief annotations, the detailed information page contains material on metabolic pathways from KEGG Orthology (KO) [18], the nucleotide sequences of the unigene, and the gene expression level (RPKM). KO identifiers, KEGG pathway names and images for the maps are shown with external links to the KEGG website. The graphical view of the directed acyclic graph for each GO term, provided in the database DAGViz [19], can be also retrieved by clicking the ‘VIEW DAG’ button in the table for brief annotations.With the ‘Keyword Search’ function, the information on unigenes can be retrieved by keyword searches against functional annotations. In the text box for keywords, users enter a keyword (e.g., lectin) or phrase (e.g., transcription factor). When they employ multiple keywords to search for unigenes, the search options for the Boolean operator ('AND Search' or 'OR Search') must be properly selected. The functional annotations obtained from three databases (nr, UniProt and GO terms) are employed by the default search function. Users can select the database (nr, UniProt and GO terms) to use for the search. As in the ‘GO Tree View’ described above, search results are shown on a gene list page with the query keyword(s) (Figure 2F). In a table, gene names are shown with brief functional annotations. The query keywords described in the brief annotations are highlighted in red font. A button ‘Download Fasta’ on the retrieved page provides the sequence data of the unigenes (Figure 2D, F).

The ‘BLAST Search’ function provides information on unigenes by using sequence similarity searches. The BLAST program package (ver. 2.2.26), which incorporates BLASTN, TBLASTN and TBLASTX [13], was implemented into A-WINGS (Figure 2F). A nucleotide or amino acid sequence(s) can be used as a query sequence(s). A query sequence can be submitted by entering it into the query box or by uploading a FASTA formatted file. Users can also simultaneously submit multiple query sequences in the FASTA format. The following search options in the BLAST programs can be set in the interactive interface: the arbitrary threshold of E-value, maximum number of alignments, word length (which controls the sensitivity), filtering option (for masking highly repetitive sequences), score matrix, and strand.

The ‘DOWNLOAD’ page provides hyperlinks for downloading files. It allows users to obtain raw data of genome sequencing reads, RNA-seq reads, unigene sequences (assembled sequences for transcriptome) and scaffolds obtained by assembling of genome sequencing reads [12].

The A-WINGS database serves as a primary bioinformation portal for the angel’s wing oyster mushroom omic information; the portal can be accessed at http://bioinf.mind.meiji.ac.jp/a-wings/. A-WINGS facilitates the identification of expressed genes and their biological functions, as well as the analysis of the mushroom’s genomes and the family *Tricholomataceae*. The database will be maintained and updated with the additional omics data as they become available. The web interfaces will be also improved for easier handling and interpretation of large-scale omics data. For example, the genome browser GBrowse [20] will be integrated into A-WINGS to make it possible to explore genomic sequences and annotations. The continual updating of omics information will further the database’s contribution to fungal research.

### Availability

A-WINGS is freely available. All questions and comments should be sent via email to achkawa@ipc.shizuoka.ac.jp and kyano@isc.meiji.ac.jp. We welcome any constructive requests for implementing additional features into A-WINGS in the future.

Project name: A-WINGS.

Project home page: http://bioinf.mind.meiji.ac.jp/a-wings/.

Operating system: Plantform independent.

Programming languages: PHP, HTML

Other requirements: None.

License: None required.

## References

[1] Matsumoto T, Nagasawa E, Fukuhara-Nakai Y (2005). Variation of ITS sequences in a natural Japanese population of *Pleurocybella porrigens*. Mycoscience.

[2] Kirk PM, Cannon PF, David JC, Stalpers JA (2001). Ainsworth & Bisby's Dictionary of the Fungi. 9th ed. CAB International.

[3] Amakura Y, Kondo K, Akiyama H, Ito H, Hatano T, Yoshida T, Maitani T (2006). Conjugated ketonic fatty acids from *Pleurocybella porrigens*. Chem Pharm Bull.

[4] Amakura Y, Kondo K, Akiyama H, Ito H, Hatano T, Yoshida T, Maitani T (2006). Characteristic long-chain fatty acid of *Pleurocybella porrigens*. Shokuhin Eiseigaku Zasshi.

[5] Sasaki H, Akiyama H, Yoshida Y, Kondo K, Amakura Y, Kasahara Y, Maitani T (2006). Sugihiratake mushroom (angel's wing mushroom)-induced cryptogenic encephalopathy may involve vitamin D analogues. Biol Pharm Bull.

[6] Hasegawa T, Ishibashi M, Takata T, Takano F, Ohta T (2007). Cytotoxic fatty acid from *Pleurocybella porrigens*. Chem Pharm Bull.

[7] Gonmori K, Yokoyama K (2009). Acute encephalopathy caused by cyanogenic fungi in 2004, and magic mushroom regulation in Japan. Chudoku Kenkyu.

[8] Takata T, Hasegawa T, Tatsuno T, Date J, Ishigaki Y, Nakamura Y, Tomosugi N, Takano F, Ohta T (2009). Isolation of *N* -acetylneuraminic acid and *N* -glycolylneuraminic acid from *Pleurocybella porrigens*. J Health Sci.

[9] Kawaguchi T, Suzuki T, Kobayashi Y, Kodani S, Hirai H, Nagai K, Kawagishi H (2009). Unusual amino acid derivatives from the mushroom *Pleurocybella porrigens*. Tetrahedron.

[10] Suzuki T, Amano Y, Fujita M, Kobayashi Y, Dohra H, Hirai H, Murata T, Usui T, Morita T, Kawagishi H (2009). Purification, characterization, and cDNA cloning of a lectin from the mushroom *Pleurocybella porrigens*. Biosci Biotechnol Biochem.

[11] Wakimoto T, Asakawa T, Akahoshi S, Suzuki T, Nagai K, Kawagishi H, Kan T (2010). Proof of the existence of an unstable amino acid: pleurocybellaziridine in *Pleurocybella porrigens*. Angew Chem Int Ed.

[12] Suzuki T, Igarashi K, Dohra H, Someya T, Takano T, Harada K, Omae S, Hirai H, Yano K, Kawagishi H (2013). A new omics data resource of *Pleurocybella porrigens* for gene discovery. PLoS One.

[13] Altschul SF, Gish W, Miller W, Myers EW, Lipman DJ (1990). Basic local alignment search tool. J Mol Biol.

[14] Pruitt KD, Tatusova T, Maglott DR (2005). NCBI Reference Sequence (RefSeq): a curated non-redundant sequence database of genomes, transcripts and proteins. Nucleic Acids Res.

[15] Apweiler R, Bairoch A, Wu CH, Barker WC, Boeckmann B, Ferro S, Gasteiger E, Huang H, Lopez R, Magrane M, Martin MJ, Natale DA, O'Donovan C, Redaschi N, Yeh LS (2004). UniProt: the universal protein knowledgebase. Nucleic Acids Res.

[16] Ashburner M, Ball CA, Blake JA, Botstein D, Butler H, Cherry JM, Davis AP, Dolinski K, Dwight SS, Eppig JT, Harris MA, Hill DP, Issel-Tarver L, Kasarskis A, Lewis S, Matese JC, Richardson JE, Ringwald M, Rubin GM, Sherlock G (2000). Gene ontology: tool for the unification of biology. The gene ontology consortium. Nat Genet.

[17] Zdobnov EM, Apweiler R (2001). InterProScan-an integration platform for the signature-recognition methods in InterPro. Bioinformatics.

[18] Mao X, Cai T, Olyarchuk JG, Wei L (2005). Automated genome annotation and pathway identification using the KEGG Orthology (KO) as a controlled vocabulary. Bioinformatics.

[19] Yano K, Aoki K, Suzuki H, Shibata D (2009). DAGViz: a directed acyclic graph browser that supports analysis of Gene Ontology annotation. Plant biotechnology.

[20] Stein LD (2013). Using GBrowse 2.0 to visualize and share next-generation sequence data. Brief Bioinform.

